# Australian rural football club leaders as mental health advocates: an investigation of the impact of the Coach the Coach project

**DOI:** 10.1186/1752-4458-4-10

**Published:** 2010-05-19

**Authors:** David Pierce, Siaw-Teng Liaw, Jennifer Dobell, Rosemary Anderson

**Affiliations:** 1 Rural Health Academic Centre, Melbourne Medical School, The University of Melbourne, 806 Mair Street, Ballarat, Victoria 3350, Australia; 2 University of New South Wales/South West Area Health Service General Practice Unit, Fairfield Hospital, PO Box 5, Fairfield, NSW 1860, Australia; 3 Familycare, PO Box 1069, Shepparton, Victoria 3632, Australia

## Abstract

**Background:**

Mental ill health, especially depression, is recognised as an important health concern, potentially with greater impact in rural communities. This paper reports on a project, Coach the Coach, in which Australian rural football clubs were the setting and football coaches the leaders in providing greater mental health awareness and capacity to support early help seeking behaviour among young males experiencing mental health difficulties, especially depression. Coaches and other football club leaders were provided with Mental Health First Aid (MHFA) training.

**Method:**

Pre-post measures of the ability of those club leaders undertaking mental health training to recognise depression and schizophrenia and of their knowledge of evidence supported treatment options, and confidence in responding to mental health difficulties were obtained using a questionnaire. This was supplemented by focus group interviews. Pre-post questionnaire data from players in participating football clubs was used to investigate attitudes to depression, treatment options and ability to recognise depression from a clinical scenario. Key project stakeholders were also interviewed.

**Results:**

Club leaders (n = 36) who were trained in MHFA and club players (n = 275) who were not trained, participated in this evaluation. More than 50% of club leaders who undertook the training showed increased capacity to recognise mental illness and 66% reported increased confidence to respond to mental health difficulties in others. They reported that this training built upon their existing skills, fulfilled their perceived social responsibilities and empowered them. Indirect benefit to club players from this approach seemed limited as minimal changes in attitudes were reported by players. Key stakeholders regarded the project as valuable.

**Conclusions:**

Rural football clubs appear to be appropriate social structures to promote rural mental health awareness. Club leaders, including many coaches, benefit from MHFA training, reporting increased skills and confidence. Benefit to club players from this approach was less obvious. However, the generally positive findings of this study suggest further research in this area is desirable.

## Background

Mental ill health is a significant burden on the Australian community [1]. The twelve month prevalence of mental disorder is about 20%; younger people are more likely to experience mental ill health than older people [2]. Whilst differences in the prevalence of depression between urban and rural settings remain a subject for debate [3], the impact of depression in rural areas may be greater. Living outside a major population centre is associated with reduced use of specific mental health services [4]. Access to mental health services is limited by the substantial inequality in urban-rural distribution of general practitioners, psychologists and psychiatrists [4]. Young men living in rural areas are less likely to seek professional help and more likely to suicide than their urban counterparts [5]. These differences may suggest a greater impact of depression on this group.

Promoting individuals' ability to connect with others in their community has been suggested as a response to depression among young rural males [6]. Social connectedness may be facilitated by providing community leaders with evidence-based mental health education and enhanced confidence to provide support to those experiencing mental ill health. In addition, addressing limited mental health literacy and stigmatising attitudes towards mental health disorders, which have been recognised as barriers to effective initial mental health responses, may also increase the level of mental health understanding and support in rural communities [7].

### Coach the Coach project

Prompted by the deaths of a number of young males from suicide in a region of rural Australia, a mental health initiative was undertaken. Using football clubs as the setting and football coaches as the leaders, supporting early help seeking behaviour among young males experiencing mental health difficulties, especially depression, was tested.

Football clubs, which are a social hub in many rural communities, were seen as an effective conduit to access young people experiencing mental health difficulties. The Coach the Coach project, with the sub-title *don't wait - talk to a mate*, resulted from this initiative. A community welfare organisation and regional football league combined to develop the project. With financial support from *beyondblue*, the Rural Health Academic Centre at The University of Melbourne assessed the impact of the project.

Mental Health First Aid (MHFA) is an established training initiative which aims to promote response to mental health problems in a similar way to more established first aid programs for physical health and medical emergencies [8]. Its use has been evaluated in rural settings [9,10] and has been reported to be linked to an increase in mental health literacy and more positive attitudes to mental health issues [11-13]. It has been used to develop mental health capacity in rural communities [14]. MHFA was selected as the most appropriate approach to develop the mental health knowledge, confidence and skills of football coaches. The program covers depressive, anxiety and psychotic disorders, with education of overlapping substance abuse [11]. A MHFA qualified instructor, who had a long association with rural football clubs, provided the training.

Coach the Coach was envisaged as an early intervention project, using MHFA to enhance the capacity of football club coaches, as local community leaders, to respond to mental health issues, especially depression. It was hoped that these key individuals may become a resource for club players. By providing a readily identifiable, respected and easily accessed source of help (football coach), it was anticipated that football club members may be empowered to respond earlier and effectively to personal or peer mental health issues before a serious mental health crisis developed [15,16]. Recognising the contribution rural sports clubs make to the social capital of rural Australia [17], the project sought to use both the established social structure and the credibility of football club leaders. It was hoped that coaches could be used as enablers to foster early help seeking behaviour especially in young rural males linked to those rural football clubs.

This paper reports on the evaluation of the Coach the Coach project undertaken during 2007 and early 2008 and discusses the provision of MHFA training in a cluster of football clubs in a rural Australian football league. The project aimed to: increase football club leaders' mental health literacy and confidence, especially about depression; promote an environment that enhances early and effective response to mental health concerns by players; and utilise rural football clubs to facilitate increased awareness of mental health issues, among those associated with football clubs and the local community. Integrated multifaceted interventions may be more effective than single interventions in promoting individual behaviour change [18]. The project therefore adopted the following linked approaches:

• Football club coaches (and other club leaders) underwent mental health first aid training (12 hrs over three weeks) using the previously established MHFA training protocol [8].

• Mental health information was provided to football club members, especially players, using both formal (e.g. specific meetings) and informal approaches (e.g. making written information material available)

• The prominent community position of rural football clubs was utilised to facilitate wider community mental health information sessions that included, where possible, a presentation on the issue of responding to depression by a high profile sportsperson.

• Awareness of mental health issues was informally promoted to football club members through the rural football league and local media.

## Methods

A mixed method approach with a before-after controlled intervention was used to assess the impact of the Coach the Coach project. Football club coaches, other club leaders and players were recruited to the assessment process through the internal communication processes of their club. In addition information about Coach the Coach came from the participating football league, and through the local media. A multifaceted approach to assessment of the impact of Coach the Coach was undertaken using the following:

### Football club leaders trained in MHFA

• Variables measured included ability to recognise depression and schizophrenia from a clinical scenario, knowledge of evidence supported treatment options, and attitudes including confidence in responding to mental health difficulties. A self-completed questionnaire that had been used in previous MHFA research [12] was used. It was administered immediately before training and six months later, providing pre-post measures. (The inclusion of vignettes depicting depression and schizophrenia reflects the importance of supporting early help seeking behaviour for these conditions [19].)

• Focus group interviews of a number of these individuals investigating their experience of the training, its impact within their club and their experience in responding to mental health difficulties.

### Football club players

• Variables measured included attitudes to depression and treatment options, and ability to recognise depression from a clinical scenario. A self-completed customised questionnaire was used. It was administered at the beginning of the project and again six months later. Although these players had not directly participated in the training, many were exposed to club information about the project and its aims, and may have attended the community mental health information sessions. Therefore, players from another football league 250km away that had not been involved in MHFA training, were recruited to complete this questionnaire at the same time as the follow-up (six month) data collection.

### Other assessment approaches

• Individual interviews of key project stakeholders, including staff of community and sporting organisations associated with the project, were undertaken at the end of the project.

• Field observations were undertaken throughout the project.

This research investigating the impact of Coach the Coach project received ethics approval from The University of Melbourne. Participant consent, in writing, was obtained before participation in the evaluation process.

For comparative data analysis a Wilcoxon signed rank test was used and for testing associations a Chi-square test was used. Statistical significance was set at p < 0.05. Interview/focus group data were transcribed, read and re-read and analysed thematically.

## Findings

### 1. Participant demographics

Thirty six football club leaders (n = 36) completed MHFA training; at least one participant came from each of the 12 clubs in the participating football league. All but one was male. The median age was 45 (range 25-64). More than half had undergone post-secondary education (trade/apprenticeship or university degree). Few, 6/36 (17%) were likely to have professional contact with young people in mental distress (health professionals, educators or law enforcement officers). Pre-training data was available from all who completed MHFA training (n = 36) and follow-up data from 66% (n = 24).

Players (n = 275) from eleven different clubs completed the initial questionnaire. The median age of these players was 21 (range 15 - 50), 23% were under 18yrs of age and 70% under 25 yrs. The follow up survey was completed by 98 players from nine different clubs - pre-post questionnaire matching was achieved for all of these players. Data collection logistics resulted in follow-up data being collected at the beginning of the following football season, a delay of two additional months. In addition, 96 questionnaires were obtained from players in the comparison football league that had not been involved in MHFA training of football club leaders.

### 2. Football club leaders (trained in MHFA)

#### (i)Recognition of mental illness

Participants' capacity to recognise depression and schizophrenia was measured by a free response to two brief scenarios typical of major depression and schizophrenia respectively. Following training, a marked improvement in capacity to recognise both depression and schizophrenia was found. See Table 1.

**Table 1:** Number of leaders trained in MHFA who correctly identified depression or schizophrenia as likely cause for symptoms of patient described in a brief clinical vignette.

	*Depression*	*Schizophrenia*
***Before MHFA training***	16/36 (44%)	8/35 (23%)
***Six months post MHFA training***	22/22 (100%)	18/23 (78%)
***Improved identification***	12/22 (55%) p = 0.001	13/23 (57%) p = 0.001
***Diminished identification***	0/22 (0%)	1/23 (4%)

Pre-post paired data on capacity to recognise depression and schizophrenia were available from 22 and 23 participants respectively. Twelve improved their capacity to recognise depression and 13 to recognise schizophrenia

#### (ii) Mental health attitudes

Participants' views about a range of responses to depression were investigated. Family doctors/GPs, counsellors, close family, close friends, psychiatrists, telephone counselling services and social workers were all regarded as helpful by more than 80% of participants. Clinical psychologists were less favourably viewed than counsellors (the respective role of each health professional was not defined in the questionnaire). Most participants did not view naturopaths as helpful. Minimal change was noted in responses between initial and follow-up measures with the exception of treating problems by self (without assistance). This approach was regarded as a harmful approach by 26/35 (74%) in the initial survey and by 22/23 (96%) in the follow up survey.

Most participants (more than 80%) positively rated the role of counselling, exercise, contact with others (getting out more), reading about others' response to depression and relaxation courses. Increased confidence that antidepressant medication, cognitive behavioural therapy (CBT) and stopping alcohol are helpful approaches to depression was reported between initial and follow-up measures. A similar change in those regarding the use of alcohol to relax as harmful was also reported. See Table 2.

**Table 2:** Leaders pre and post MHFA training agreement with statements about depression management strategies (expressed as percentages).

	Pre MHFA training	Post MHFA training	
***Antidepressants helpful treatment for depression***	39	83	p = 0.01
***CBT helpful treatment for depression***	68	88	P = 0.20
***Stopping alcohol helpful for depression***	82	92	P = 0.25
***Using alcohol to relax harmful for depression***	26	46	P = 0.12

Participants' confidence in helping someone with a mental health problem increased markedly. Matched paired data on mental health confidence was available from 24 participants, with most (16/24) reporting increased confidence and few (3/24) reporting decreased confidence. Before training, more than one third reported their confidence level as being 'not at all' or 'a little bit'. Six months later, no participant reported these low levels of confidence. See Table 3.

**Table 3:** Leaders confidence in helping someone with a mental health problem following MHFAtraining.

Increased confidence	Decreased confidence	Unchanged confidence	
16/24	3/24	5/24	P = 0.004

#### (iii) Training and post training experience

Eleven football coaches and club leaders drawn from a range of clubs participated in one of two focus groups that were conducted by a member of the research team. No players participated in the focus groups. Key emergent themes included: building on existing (mental health) experience; development of empowerment; and early application of new mental health skills. Each of these themes is now considered.

Building on existing mental health experience

MHFA training was seen as building upon previous experience and developing existing knowledge, rather than learning from scratch. Past personal experience was a motivating factor to learn more and feel more able to contribute in the future, not just with footballers but in the wider community.

*I was very excited, because I have been involved with people who have gone through hard times. And so to learn, to be more understanding to see signs more, not just with the footballers but in the community*.... Participant 04

*I knew a bit around the edges but it gave me a lot of ... a lot of context, which I wouldn't have thought about*. Participant 01

A number of participants saw MHFA training as fulfilling the many social responsibilities of rural football clubs. Initial apprehension turned to enthusiasm when the opportunity to contribute to the lives of young footballers became clear.

*I was excited about the opportunity ... being involved with Under 18's it might help my understanding about what planet they are on*. Participant 02

Empowerment and early application of skills

Many reported going back to their clubs enthused and empowered by the experience of the training. A number reported an early positive impact on the players in their clubs, utilising the training material. Some utilised the written material presented as part of the training program in their clubs, others spoke more directly to their players.

*I heard them *[players]*talking about it in the showers, so it obviously provoked them ....So if nothing else the players and the people were aware that we were serious about *[mental health]. Participant 01

Not all found communicating about mental health issues to be easy; but they reported persisting in a number of ways and succeeding.

..... *there are posters above every urinal; in every female toilet beside the toilet paper there is something on depression or anxiety or disorders and I think largely those posters remain intact which indicates that they are appreciative I believe of the message because most times they get vandalised*. Participant 05

Specific events were reported which reflected participants feeling empowered by the training to act in a more effective way. A club leader, reflecting on an episode in which a player indicated he was going to kill himself, commented that had this event occurred before the training his response would have been:

*Yeah I probably would have felt embarrassed and walked away and thought, "Deal with it." And he would have dealt with it I am convinced about that*. Participant 06

Participants were excited and perhaps surprised by the degree of empowerment they had achieved. Empowerment to respond constructively to mental health problems was not limited to the football club; some participants reported also having had a positive impact on distressed individuals in their local community (see quote by Participant 04 above).

### 3. Football club players

#### (i) Attitudes to a depressed person

In the setting of a brief scenario of a young person with depression most players (70%) in the initial questionnaire did not regard depression as a sign of personal weakness and many (60%) did not feel a depressed young person could snap out of 'their problem'. In addition most (65%) disagreed with the approach of not telling anyone if they were experiencing depression. These findings were the same in the initial and follow-up questionnaires and in those from the comparison football league. In the follow-up questionnaire many players (45%) were less likely than in the initial survey to regard a young person with depression as dangerous (p = 0.10).

#### (ii) Attitudes to seeking help (if depressed)

Players indicated they were most likely to seek help from a family member, a mate, or a GP and were least likely to seek help from telephone counselling, posters, pamphlets or a priest. Given the age group involved the finding that only 1 in 4 would also seek assistance from the internet was unexpected. See Table 4.

**Table 4:** Sources of help football club players would use if depressed - percentage of players reporting that they would seek help from each individual source.

Source of help	Intervention League Initial	Intervention League Follow-up		Comparison League	
***A member of your family***	79	77	P = 0.33*	64	P = 0.38**
***A mate***	75	73	P = 0.28*	68	P = 0.49**
***Football club leader***	41	41	P = 0.52*	26	P = 0.62**
***A minister/priest***	10	8	P = 0.08*	11	P = 0.04**
***Telephone counselling***	17	22	P = 0.39*	15	P = 0.60**
***Internet***	23	25	P = 0.28*	25	P = 0.74**
***Poster/pamphlets***	24	23	P = 0.42*	21	P = 0.16**
***GP/family doctor***	62	63	P = 0.54*	66	P = 0.24**

* Comparing initial intervention league data with follow-up intervention league data

** Comparing follow-up intervention league data with comparison league data

Data were collected from comparison football league players at the same time as follow-up data from the intervention football league

Approximately one in three players reported that they were still at school. Few in this group indicated that established mental health support avenues related to schools, in particular school counsellors and teachers, were perceived as a likely source of help.

### 4. Key stakeholder views

A number of issues were identified by key stakeholders as influencing the impact of the project. The key stakeholders regarded football clubs as influential in rural communities and football coaches/club leaders as frequently having achieved success and recognition among club players. Utilising these dual influences was seen as an appropriate way to drive the knowledge, awareness and attitudinal changes needed to effectively address mental health issues in rural communities.

The community depression information sessions facilitated by participating clubs were regarded as a valuable part of the project. In addition to promoting awareness of depression and addressing the frequently reported associated stigma, these sessions announced both to the club members and the local community that individuals, trained in MHFA were available as a contact and for support. This was emphasised in the following comment made by one stakeholder:

[You]*can't have mental health skilled people running around in stealth; people need to know who they are in the clubs*.

Wide variation in strength and functional structure of football clubs was reported and felt to be important although no broadly applicable formula for success with a project such as Coach the Coach in these clubs was suggested. Finally, the project was reported to be more time demanding than originally anticipated. One participant noted the need for 'invisible time', referring to the time required to develop links, promote confidence, provide support and facilitate networking.

## Discussion

Previous research focusing on the development of football club leaders' mental health skills, including the use of MHFA training, linked with the associated use of Australian rural football clubs to promote early response to mental health concerns, is limited. This study builds upon recently reported Australian research, which focused on mental health literacy training for junior sporting clubs [20], by including measures of participating football club players' responses and by undertaking delayed follow up six months after completion of MHFA training. Football clubs were identified as established and influential in the social dynamic of many rural communities [17]. The approach used in this project avoided the need to develop a new structure in rural communities to facilitate mental health promotion among the principal target group, young rural males associated with football clubs. The positive findings of this study suggest that this model may be relevant in similar settings.

Football club leaders who were trained in MHFA demonstrated increased capacity to recognise from vignettes the features of depression and schizophrenia some six months after completion of MHFA training. Similarly their expressed confidence to assist a person experiencing a mental health problem improved. Increased mental health knowledge and confidence was expressed as empowerment to help those experiencing mental ill health. By building on football club leaders' existing skills and addressing specific issues including what to say to someone experiencing mental ill health, the training increased participants' capacity to respond to mental health issues. Increasing key individuals' awareness and knowledge of mental health issues has the potential to be a new community resource with participants not limiting their assistance to those directly associated with the football club. However, more research investigating positive community benefits beyond those who are trained is needed.

All football club leaders participating in this project were volunteers, albeit potentially encouraged by their club. All initial participants completed the MHFA training program. This research supports volunteer participants in such training. It is unclear if an obligatory approach to participation in training would have resulted in the same outcome. Henderson et al [21] have reported attitudes and skill development of high school students who were mandated to participate in community service compared to those who volunteered their time. Little difference was found between the two cohorts. Currently it is recommended that sporting coaches undertake general first aid training as part of their overall training [22]. Given the response of the coaches and other club leaders to mental health training reported in this paper, sporting code leaders may consider the inclusion of some mental health first aid training, especially in rural areas. The effectiveness of such training, especially if it was not voluntary (e.g. as a mandatory requirement for credentialing as a sports club leader) may be the subject of important future research.

The authors were surprised by the finding that most young players in the current study reported that they were unlikely to seek assistance from the internet. It is assumed that most young people are computer savvy [23]. The concept that this cohort may not access the internet for assistance on mental health concerns seems counterintuitive. Significant resources are allocated each year to develop educational material, including online material, designed to combat mental ill health [24,25]. This study suggests we should question any automatic assumptions that this approach may readily reach the at risk population of males aged 15-25 years. The finding of this study that this group may be unwilling to use the internet, telephone counselling services and written material for mental health information should be further investigated.

Many players from the participating football league reported that, if depressed, they would use help from a family member or a mate. Similar findings were reported from those in the comparison football league. It is unclear if the mental health training of club leaders influenced players to connect with others. The sample size was small and this real life study could not control for outside influences. Further research should be undertaken to ascertain if the benefits of MHFA (or similar) training are experienced by others in addition to those who undertake the course.

This study focused on the assessment of the more structured aspects of the project. Measurement of less structured components, such as increased awareness of mental health issues related to club, community publicity and written material of mental health information displayed in changing rooms was limited. Focus group and key stakeholder interviews suggested these aspects of the project may have been associated with greater impact than initially anticipated. The potential impact of non-structured aspects of similar projects should be considered in future research.

Early in the implementation phase of the project it was decided to incorporate mental health information evenings at a number of the participating clubs. This decision may have been influenced by the positive response to the project of many clubs and a desire to capitalise on the momentum generated by the project. These evening meetings aimed to recognise and publicise those who had been trained in MHFA and were now available as resources for those experiencing mental health difficulties. These meetings aimed to confront the stigma of depression, using a high profile sportsperson speaking of their experience of depression. The late inclusion of these evenings in the project meant that available data on the impact of these sessions were limited. However, the reported observations of key stakeholders suggested that they may have been a valuable component of the project and as such should be investigated in further research. It is unlikely that such meetings, using the football club as the springboard to support information communication and encouragement of behaviour change (early response to mental health issues) would have the same impact if undertaken in isolation without the priming effect of other aspects of the overall project.

## Limitations

This study was undertaken in a rural area; a number of the findings may be specific to rural settings and possibly less applicable in larger population centres with potentially different dynamics related to the influence of the football club on mental health programs. The high attrition rate of players is a limitation of this study. This attrition meant that detection of significant change in the views of players over the duration of the project was difficult to recognise. Attrition of players from the study may have been less had the project been completed within a single football season.

## Conclusions

This study suggests that football clubs may be appropriate social structures to act as the base for initiatives to increase mental health capacity in rural communities. It suggests that football club leaders trained in mental health first aid feel more confident responding to a person who may be experiencing mental ill health. Further investigation of any associated impact upon club players and the wider rural community should be undertaken. It remains unclear if educating and empowering in mental health issues a limited number of sports club leaders empowers others with whom they interact.

## Competing interests

The authors declare that they have no competing interests.

## Authors' contributions

DP, TL and JD all contributed to the study conception and implementation. DP and RA contributed to data analysis. DP wrote the initial draft of the paper and RA contributed to manuscript editing. All authors approved the final manuscript.

## References

[B1] Moore M Shaw J Grant B Braddock D Institutional mental health services in Australia 1997-98: first report on the national minimum data set - institutional mental health care 2000 Canberra: Australian Institute of Health and Welfare

[B2] Treatment Management Protocol Management of Mental Disorders 2004 4 Sydney: World Health Organisation Collaborating Centre for Evidence in Mental Health Policy

[B3] Judd F Jackson H Komiti A Murray G Hodgins G Fraser C High prevalence disorders in urban and rural communities Aust N Z J Psychiatry 2002 36 104 113 10.1046/j.1440-1614.2002.00986.x 11929446

[B4] Fraser C Judd F Jackson H Murray G Humphreys J Hodgins G Does one size fits all? Why the mental health of rural Australians requires further research Aust J Rural Health 2002 10 288 295 10.1046/j.1440-1584.2002.00463.x 12472610

[B5] Caldwell T Jorm A Dear K Suicide and mental health in rural, remote and metropolitan areas of Australia Med J Aust 2004 181 7 Suppl S10 S14 15462636 10.5694/j.1326-5377.2004.tb06348.x

[B6] Wainer J Chester J Rural mental health: neither romanticism nor despair Aust J Rural Health 2000 8 141 147 10.1046/j.1440-1584.2000.00304.x 11249401

[B7] Jorm A Blewitt K Griffiths K Kitchener B Parslow R Mental health first aid: responses of the public BMC Psychiatry 2005 5 9 10.1186/1471-244X-5-9 15694008 PMC549043

[B8] Mental Health First Aid 2007 http://www.mhfa.com.au [cited 25Aug2007]

[B9] Jorm A Kitchener B O'Kearney R Dear K Mental health first aid training of the public in a rural area: a cluster randomized trial BMC psychiatry 2004 4 33 10.1186/1471-244X-4-33 15500695 PMC526774

[B10] Hossain D Gorman Eley R Enhancing the knowledge and skills of Advisory and Extension Agents in mental health issues of farmers Australas Psychiatry 2009 17 1 Suppl S116 S120 10.1080/10398560902948365 19579123

[B11] Kitchener B Jorm A Mental health first aid training of the public: evaluation of effects on knowledge, attitudes and helping behaviour BMC Psychiatry 2002 2 10 10.1186/1471-244X-2-10 12359045 PMC130043

[B12] Kitchener B Jorm A Mental health first aid in a workplace setting: a randomized controlled trial BMC Psychiatry 2004 4 23 10.1186/1471-244X-4-23 15310395 PMC514553

[B13] Kitchener B Jorm A Mental health first aid training: review of evaluation studies Aust N Z J Psychiatry 2006 40 6 8 16403032 10.1080/j.1440-1614.2006.01735.x

[B14] Sartore G Kelly B Stain H Fuller J Fragar L Tonna A Improving mental health capacity in rural communities: Mental health first aid delivered in drought-affected rural New South Wales Aust J Rural Health 2008 16 313 318 10.1111/j.1440-1584.2008.01005.x 18808491

[B15] Allen N Hetrick S Simmons J Hickie I Early intervention for depressive disorders in young people: the opportunity and the (lack of) evidence Med J Aust 2007 187 7 Suppl S15 S17 17908018 10.5694/j.1326-5377.2007.tb01329.x

[B16] Kelly C Jorm A Wright A Improving mental health literacy as a strategy to facilitate early intervention for mental disorders Med J Aust 2007 187 7 Suppl S26 S30 17908021 10.5694/j.1326-5377.2007.tb01332.x

[B17] Mugford S The status of sport in rural and regional Australia Sports Industry Australia 2001 http://www.qqsr.com.au/RuralSportReport.pdf [cited 23Jan09]

[B18] Davies D Thomson M Oxman AD Haynes R Changing physician performance: a systematic review of the effect of continuing medical education strategies JAMA 1995 274 9 700 705 10.1001/jama.274.9.700 7650822

[B19] Jorm A Mental health literacy: Public knowledge and beliefs about mental disorders Br J Psychiatry 2000 177 396 401 10.1192/bjp.177.5.396 11059991

[B20] Bapat S Jorm A Lawrence K Evaluation of a mental health literacy training program for junior sporting clubs Australas Psychiatry 2009 17 475 479 10.1080/10398560902964586 20001370

[B21] Henderson A Brown SD Pancer S Ellis-Hale K Mandated community service in high school and subsequent civic engagement: The case of the "double cohort" in Ontario, Canada J Youth Adolesc 2007 36 849 60 10.1007/s10964-007-9207-1

[B22] NAB Auskick Manual 2009 Melbourne: Australian Football League 190 9

[B23] Gross EF Adolescent Internet use: What we expect, what teens report J Appl Dev Psychol 2004 25 633 49 10.1016/j.appdev.2004.09.005

[B24] Spence SH Donovan CL March S Gamble A Anderson R Prosser S Kercher A Kenardy J Online CBT in the treatment of child and adolescent anxiety disorders: Issues in the development of BRAVE-ONLINE and two case illustrations Behav Cogn Psychother 2008 36 411 30 10.1017/S135246580800444X

[B25] MacLeod M Martinez R Williams C Cognitive behaviour therapy self help: Who does it help and what are its drawbacks? Behav Cogn Psychother 2009 37 61 72 10.1017/S1352465808005031 19364408

